# Different Origins of Newcastle Disease Virus Hemagglutinin-Neuraminidase Protein Modulate the Replication Efficiency and Pathogenicity of the Virus

**DOI:** 10.3389/fmicb.2017.01607

**Published:** 2017-08-23

**Authors:** Ji-hui Jin, Jin-long Cheng, Zi-rong He, Ying-chao Ren, Xiao-hui Yu, Yang Song, Hui-ming Yang, Yan-ling Yang, Tong Liu, Guo-zhong Zhang

**Affiliations:** ^1^Key Laboratory of Animal Epidemiology of the Ministry of Agriculture, College of Veterinary Medicine, China Agricultural University Beijing, China; ^2^Diagnostic and Research Center of Livestock and Poultry Epidemic Diseases, China Agricultural University Beijing, China

**Keywords:** Newcastle disease virus, hemagglutinin–neuraminidase protein, reverse genetics, biological activity, replication, pathogenicity

## Abstract

To investigate the exact effects of different origins of Newcastle disease virus (NDV) hemagglutinin-neuraminidase (HN) protein to the biological characteristics of the virus, we systematically studied the correlation between the HN protein and NDV virulence by exchanging the HN of velogenic or lentogenic NDV strains with the HN from other strains of different virulence. The results revealed that the rSG10 or rLaSota derivatives bearing the HN gene of other viruses exhibited decreased or increased hemadsorption (HAd), neuraminidase and fusion promotion activities. *In vitro* and *in vivo* tests further showed that changes in replication level, tissue tropism and virulence of the chimeric viruses were also consistent with these biological activities. These findings demonstrated that the balance among three biological activities caused variation in replication and pathogenicity of the virus, which was closely related to the origin of the HN protein. Our study highlights the importance of the HN glycoprotein in modulating the virulence of NDV and contributes to a more complete understanding of the virulence of NDV.

## Introduction

Newcastle disease (ND) is a constant threat to the poultry industry worldwide and has caused severe economic losses. The causative pathogen is Newcastle disease virus (NDV), which is a member of the genus *Avulavirus* in the family *Paramyxoviridae* (Mayo, 2002). NDV strains are classified into three major pathotypes: lentogenic, mesogenic, and velogenic, based on their pathogenicity in chickens. NDV can be classified into two classes based on genome length and the sequence of the F gene. Class I viruses are less genetically diverse, are generally present in wild waterfowl, and are of low virulence (Dimitrov et al., 2016). Class II can be further divided into nine genotypes (Munir et al., 2012). Among these, genotype VII NDVs are the predominant strains isolated throughout the world in recent years (Miller et al., 2009). Genotype VI viruses, sometimes referred to as pigeon paramyxovirus serotype 1, are generally of moderate virulence (Dortmans et al., 2009). Genotype II within class II includes isolates of high and low virulence, and are often used as vaccine strains. Strains of avian paramyxovirus type 2 (APMV-2) is much less virulent and have been associated with asymptomatic to mild respiratory diseases in chickens (Alexander, 1982).

Newcastle disease virus has a non-segmented, single-stranded, negative-sense RNA genome of 15,186, 15,192, or 15,198 nucleotides in length. The genome encodes six structural proteins: nucleoprotein (NP), phosphoprotein (P), matrix protein (M), fusion protein (F), hemagglutinin-neuraminidase (HN) and large polymerase protein (L), transcribed from six genes in the following order: 3′-NP-P-M-F-HN-L-5′. RNA editing of the P protein produces additional non-structural proteins V and W (Miller and Koch, 2013). NDV infection is initiated by receptor recognition and binding to the host cell surface, followed by fusion, which is accomplished by the interaction of F and HN proteins (Connaris et al., 2002).

Though the amino acid sequence at the F protein cleavage site has been identified as the primary determinant of NDV virulence, the NDV HN protein plays an important role in viral invasion and maturation, and is present on the surface of virions and infected cells (Peeters et al., 1999; Panda et al., 2004; Jin et al., 2016). HN is a type II homotetrameric glycoprotein consisting of an N-terminal transmembrane domain and an ectodomain, which has a globular head perched on top of a membrane-anchored stalk domain (Yuan et al., 2012). HN is a multifunctional molecule with three distinct activities: receptor binding, neuraminidase (NA) activity and fusion promotion (Mirza et al., 1994; Melanson and Iorio, 2004). The globular head includes two receptor-binding sites, site I is associated with receptor binding and neuraminidase activity, site II is involved in receptor binding and fusion (Crennell et al., 2000; Iorio et al., 2001; Zaitsev et al., 2004; Jin et al., 2016). In addition, the stalk domain promotes membrane fusion through its interaction with the F protein (Melanson and Iorio, 2004; Porotto et al., 2012). The HN protein has been shown to contribute greatly to NDV pathogenesis (Huang et al., 2004; Kim et al., 2011; Cornax et al., 2013).

Several studies have investigated the contribution of the HN gene to NDV virulence and tropism by exchanging genes between strains (Huang et al., 2004; Oldoni et al., 2005; Wakamatsu et al., 2006; Paldurai et al., 2014). Although these studies have increased our understanding of the role of HN in NDV virulence, some of the results have been controversial and conflicting. The HN from a lentogenic virus (LaSota) inserted into a virulent backbone (Beaudette C; BC) caused dissemination of virus in a manner similar to wild type virulent virus (BC) (Oldoni et al., 2005). On the contrary, the LaSota HN within the BC backbone decreased disease severity and pathogenicity indices (Huang et al., 2004; Wakamatsu et al., 2006). Presence of HN from virulent virus BC inserted into a LaSota backbone caused dissemination of virus in a manner similar to BC (Huang et al., 2004; Oldoni et al., 2005). However, the BC HN within the LaSota backbone did not change either severity of disease in chickens or pathogenicity indices (Wakamatsu et al., 2006). Furthermore, the HN genes of a velogenic NDV strain GB Texas (GBT) and the mesogenic BC were exchanged, but the tropism and virulence of the chimeric recombinant viruses were not obviously altered (Paldurai et al., 2014).

In this study, we conducted a systematic study of the contribution of the HN protein to NDV virulence and pathogenesis by replacing the complete HN open reading frame (ORF) of a velogenic genotype VII SG10 strain and a lentogenic genotype II LaSota strain, with HN proteins of different origins. The complete HN ORF of strains BJ (a mesogenic genotype VI NDV), LaSota, HB (a lentogenic class I NDV) and Yucaipa (a lentogenic APMV-2 strain) were inserted into the rSG10 backbone and the complete HN ORF of rLaSota was replaced with the corresponding HN ORF of strains SG10, BJ, HB and Yucaipa. A total of seven chimeric viruses, plus the two parent strains, were recovered. We evaluated the viral replication in DF-1 cells and 3-week-old specific-pathogen-free (SPF) chickens, virulence in 9-day-old SPF chicken eggs, 1-day-old SPF chicks and 6-week-old SPF chickens, pathogenicity in 3-week-old SPF chickens, and HN biological activities at viral and protein levels *in vitro*.

## Materials and Methods

### Animals and Ethics Statement

Specific-pathogen-free chickens and embryonated eggs were purchased from Merial Vital Laboratory Animal Technology Co., Ltd. (Beijing, China). All birds were kept in isolators and the animal rearing facilities were approved by the Administration Committee of Laboratory Animals under the auspices of the Beijing Association for Science and Technology (approval ID SYXK [Jing] 2013-0013). The experimental protocol, including the possibility of animal death without euthanasia, was specifically considered and approved by the Animal Welfare and Ethical Censor Committee at China Agricultural University (CAU approval number 1605–01).

### Cells and Viruses

Baby hamster kidney (BHK-21) cells stably expressing T7 RNA polymerase (BSR T7), an African green monkey kidney cell line (Vero) and a chicken embryo fibroblast cell line (DF-1) were all grown in Dulbecco’s modified eagle medium (DMEM, Gibco, Grand Island, NY, New York) containing 10% (v/v) fetal bovine serum (FBS, Gibco), and maintained in DMEM containing 2% FBS at 37°C in an incubator (Thermo Forma, Marietta, OH, United States) under 5% CO_2_. Recombinant NDV strains rSG10 and rLaSota were generated in our laboratory (Liu M.M. et al., 2015; Yu et al., 2017). The velogenic genotype VII NDV strain SG10, mesogenic genotype VI NDV strain BJ, lentogenic genotype II NDV strain LaSota, lentogenic class I NDV strain HB and lentogenic APMV-2 strain Yucaipa were propagated in 9-day-old SPF chicken eggs.

### Construction of Full-Length Chimeric SG10/LaSota Antigenomic cDNAs and Generation of Chimeric Viruses

**FIGURE 1:**
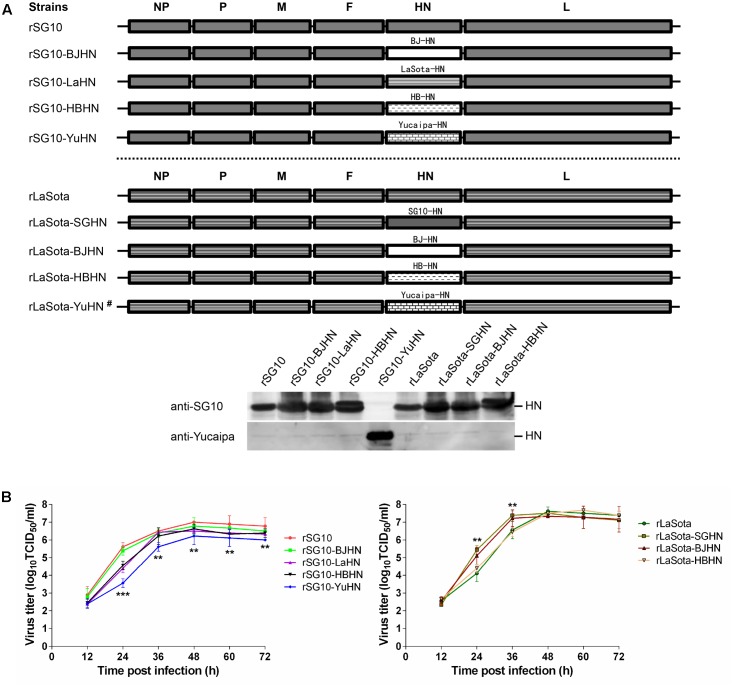
Recovery, identification, and growth characterization of recombinant viruses. **(A)** Construction of full-length antigenomic cDNAs of rSG10 or rLaSota in which the complete HN glycoprotein was replaced with that of the other virus. The complete HN ORFs of strains BJ, LaSota, HB, and Yucaipa were inserted into the rSG10 backbone in place of the native HN gene. Similarly, the complete HN ORFs of strains SG10, BJ, HB, and Yucaipa were placed into a full-length antigenomic cDNA of the corresponding rLaSota HN ORF. Seven chimeric viruses were successfully recovered, and the chimeric virus rLaSota-YuHN that could not be rescued is indicated with #. Western blot analysis was used to identify HN proteins of recombinant viruses using polyclonal antibody raised against the NDV SG10 or Yucaipa strains. **(B)** *In vitro* growth kinetics of parental and chimeric viruses in DF-1 cells. DF-1 cells were infected with chimeric or parental viruses at an MOI of 0.01, and assayed as described in the Methods. Each bar represents the mean and standard deviation of three independent experiments. Asterisks indicate the significance of the difference between the chimeric and parental viral titers. P values were calculated with Tukey’s test (95% confidence levels). ^∗∗^*p* < 0.01, very significant; ^∗∗∗^*p* < 0.001, extremely significant.

The HN ORF of genotype VI NDV strain BJ (571 aa), genotype II NDV strain LaSota (572 aa), class I NDV strain HB (616 aa) and APMV-2 strain Yucaipa (580 aa) were individually placed into the full-length antigenomic cDNA of strain rSG10 (571 aa) in place of the corresponding NDV HN ORF via the presence of unique restriction enzyme sites with the Seamless Assembly Cloning Kit (Invitrogen, Carlsbad, CA) (**Figure 1A**). Firstly, the full-length antigenome of rSG10 was digested with *Sal*I and *Mlu*I to generate a single DNA fragment without the HN gene. Then, the HN ORFs of BJ, LaSota, HB and Yucaipa were generated by PCR amplification as DNA fragments each flanked by 5′ and 3′ UTRs of the rSG10 HN gene. To maintain the genome length in multiples of six nucleotides, additional nucleotides were introduced at the 3′ UTR of the HN gene as necessary. The other two DNA fragments with homologous ends, one extending from the *SalI* site to the HN initiation codon and the other from the HN termination codon to the *MluI* site, were generated by PCR amplification with compatible primers. Finally, these four DNA fragments with overlapping homologous ends of various lengths were effectively joined together using the cloning kit. The complete HN ORFs of strains SG10, BJ, HB and Yucaipa were inserted into the rLaSota backbone in place of the corresponding rLaSota sequence using the same strategy (**Figure 1A**). The full-length antigenome of rLaSota was digested with *XbaI* and *HindIII* to generate a single DNA fragment without the HN gene. All the replaced regions in the full-length cDNAs were sequenced to confirm the presence of the desired genes. The resulting full-length plasmids of rSG10 and rLaSota encoding the different HN proteins were used to recover the recombinant viruses.

Virus rescue was performed as described previously (Liu M.M. et al., 2015; Yu et al., 2017). Briefly, the respective helper plasmids individually encoding the NP, P and L proteins of either rSG10 or rLaSota were used along with their respective full-length cDNAs in transfected BSR T7 cells. Cells transfected with rLaSota chimeric cDNAs were cultured in medium with 5 μg/ml *N*-tosylphenylalanine chloromethyl ketone (TPCK)-treated trypsin (Sigma–Aldrich, St. Louis, MO, United States) (Madansky and Bratt, 1978). Four days after transfection, the cell culture supernatant was inoculated into 9-day-old embryonated SPF chicken eggs via the allantoic cavity route. Recovery of the virus was confirmed by hemagglutination (HA) assay. Total RNA was extracted from NDV-positive allantoic fluid with TRIzol reagent (Invitrogen) according to the manufacturer’s instructions. The sequences of the HN genes in the recovered chimeric viruses were confirmed by reverse transcription (RT) PCR and Western blot analysis.

### Construction of HN and F Protein Expression Plasmids

The HN genes of strains BJ, LaSota, HB, Yucaipa and F gene of LaSota were amplified from full-length cDNA clones as described above and were inserted into pCI-neo (pCI) plasmid (Promega, Madison, WI, United States) between the *EcoRI* and *XbaI* restriction sites. The obtained plasmids were named pCI-BJHN, pCI-LaHN, pCI-HBHN, pCI-YuHN and pCI-LaF. The plasmids pCI-SGHN and pCI-SGF were generated in our laboratory as described previously (Jin et al., 2016).

### Western Blot Analysis

Total protein lysates were extracted from infected or transfected cells with ice-cold RIPA lysis buffer (1% sodium deoxycholate, 1% Triton X-100, 50 mM Tris [pH 7.4], 150 mM NaCl). Cellular proteins were separated through 10% sodium dodecyl sulfate-polyacrylamide gel electrophoresis and transferred to a polyvinylidene difluoride (PVDF) membrane (Amersham Biosciences, Freiburg, Germany). Each PVDF membrane was blocked with 5% (w/v) skim milk and 0.1% Tween 20 in Tris buffered saline (TBST) and then incubated with a primary antibody at 4°C overnight. Primary antibodies were SG10, Yucaipa polyclonal serum or anti-SG10 F rabbit polyclonal antiserum (both diluted 1:100), β-actin mouse monoclonal antibody with a dilution 1:1000 (Beyotime Biotechnology, Beijing, China). After being washed with TBST, the membranes were incubated with corresponding horseradish peroxidase (HRP)-conjugated anti-chicken, anti-rabbit or anti-mouse antibody at a 1:10,000 dilution for 1 h (Bioss Biotechnology, Beijing, China). Presence of HRP was detected using a Western Lightning chemiluminescence kit (CWBIO, Beijing, China). Protein bands were normalized to β-actin and quantified by densitometry using ImageJ software (National Institute of Mental Health, Bethesda, MD, United States).

### Virus Growth Kinetics

The growth kinetics of nine NDVs, including the rSG10, rLaSota and their chimeric viruses were evaluated under multiple-cycle growth conditions in DF-1 cells. Cells in duplicate wells of six-well plates were infected with NDVs at a multiplicity of infection (MOI) of 0.01 plaque forming units (PFU)/cell. After 1 h of adsorption, the cells were washed with DMEM and then incubated with DMEM containing 2% FBS at 37°C in 5% CO_2._ The media of cells infected with rLaSota and its chimeric viruses were supplemented with 5 μg/ml TPCK-treated trypsin. Supernatants were collected at 12 h intervals until 72 h post-infection (hpi), and the viral titers were quantified in DF-1 cells and expressed as median tissue culture infective doses (TCID_50_)/ml, using the endpoint method (Reed and Muench, 1938). Cells infected with rLaSota and its chimeric viruses were identified by indirect immunofluorescence assay (IFA). In brief, at 72 hpi, cells were fixed with cold methanol for 20 min and incubated with anti-LaSota serum at 37°C for 1 h. Then the cells were washed three times with phosphate-buffered saline (PBS) and goat anti-chicken IgG-FITC (Sigma–Aldrich) was added to each well. Plates were incubated at 37°C for 1 h and washed three times with PBS. Plates were examined on an inverted fluorescence microscope.

### Virulence of Recovered Viruses

The virulence of rSG10, rLaSota and their chimeric viruses was determined with standard virulence tests for NDV: the mean death time (MDT) in 9-day-old SPF embryonated chicken eggs, the intracerebral pathogenicity index (ICPI) in 1-day-old chicks and the intravenous pathogenicity index (IVPI) in 6-week-old chickens. All tests were performed according to previously published methods (Khattar et al., 2009).

### Viral Pathogenicity in Chickens

To evaluate the pathogenicity of the rSG10-YuHN, rLaSota-SGHN and their parent strains (rSG10 and rLaSota), groups of 20 (10 for sampling and 10 for clinical observation) 3-week-old SPF chickens were inoculated with 10^5^ 50% egg infectious dose (EID_50_) of virus per bird by the oculonasal route. The birds were observed daily and scored as follows for clinical signs for 14 days post-infection (dpi): 0, healthy; 1, sick; 2, wing drop/paralysis/torticollis/incoordination; 3, prostration; 4, dead. Survival was monitored until 14 dpi. Two birds from each group were euthanized at 1, 3, 5, 7 dpi, and brain, trachea, lung, spleen, duodenum and caecum were collected for virus titration. Samples collected at 3 dpi were fixed in 10% buffered formalin for histopathology. For virus titration, tissue samples were homogenized in PBS containing antibiotics and the supernatant was serially diluted 10-fold and inoculated in DF-1 cells, in duplicate wells per dilution. The virus titers were determined by IFA as described above and the TCID_50_ per 100 μg of tissue was calculated (Reed and Muench, 1938). For histopathology, the fixed tissues were routinely embedded in paraffin wax, 5 μm sections were prepared for hematoxylin and eosin staining and examined for lesions using light microscopy.

### Hemadsorption (HAd) Assay

Confluent monolayers of Vero cells in 24-well plates were infected with 0.1 MOI of virus. Cells were washed with cold PBS at 24 hpi and then overlaid with a 2% (v/v) suspension of CRBCs at 4°C for 30 min (Li et al., 2004). Unbound CRBCs were washed with ice-cold PBS and the CRBCs bound to the virus-infected cells were lysed with an RBC lysis solution (17mM Tris-HCl, 0.145 M NH_4_Cl). The released hemoglobin was measured with a Spectramax M5 ELISA reader at 549 nm (Molecular Devices, Sunnyvale, CA, United States). The HAd assay investigating HN at the protein level was performed with monolayers of Vero cells transfected using 0.5 μg each of pCI-HN plasmids (pCI-SGHN, pCI-BJHN, pCI-LaHN, pCI-HBHN and pCI-YuHN) as described previously.

### NA Assay

The NA activity was determined using a modification of an assay described previously (Potier et al., 1979). Vero cell monolayers were infected with NDVs at 0.1 MOI. At 24 hpi, the cells were incubated at 37°C for 30 min with 30 μl of substrate mix (one volume of 325 mM 2-*N*-morpholinoethanesulfonic acid [MES; pH 6.4], two volumes of 0.5 mM 2′-(4-methylumbelliferyl)-α-D-*N*-acetylneuraminic acid [MUN; Sigma], and three volumes of 10 mM calcium chloride) per well. The reaction was terminated using 0.014 M sodium hydroxide in 83% (v/v) ethanol. Fluorescence intensity was measured at an emission wavelength of 450 nm and an excitation wavelength of 360 nm with a Spectramax M5 ELISA reader (Molecular Devices). The NA assay investigating HN at the protein level was performed with monolayers of Vero cells transfected with the same expression plasmids utilized in HAd assay.

### Fusion Index Assay

The fusogenic abilities of the rSG10 and its chimeric viruses were examined in Vero cells (Kim et al., 2011). Six-well plates seeded a day earlier at 2.0 × 10^6^ cells were infected with NDVs at 0.1 MOI and maintained in DMEM with 2% FBS at 37°C under 5% CO_2_. After monitoring cytopathic effect for 48–72 h, the cells were washed once with 0.02% EDTA, and subsequently incubated for 2 min with 1 ml of EDTA at room temperature. The cells were washed with PBS and fixed for 20 min with methanol at room temperature, and then stained with hematoxylin-eosin. Fusion was quantitated by the fusion index, i.e., the ratio of the total number of nuclei to the number of cells in which these nuclei were observed. The fusogenic properties of rLaSota and its chimeric viruses were examined in DF-1 cells and the medium was supplemented with 5 μg/ml TPCK-treated trypsin. The fusion index assay at the protein level was performed with monolayers of Vero cells co-transfected using 1 μg each of pCI-HN and pCI-SGF. Co-transfection of pCI-HN and pCI-LaF was performed in DF-1 cells with 5 μg/ml TPCK-treated trypsin. The HAd, NA and fusion index values for all the viruses or HN proteins were expressed as percentages of the values for the parental viruses (rSG10, rLaSota) or the expressed HN and F protein (pCI-SGHN, pCI-SGHN/SGF, pCI-LaHN/LaF), which were considered to be 100%.

### Hemolytic Assessment

The hemolytic activities were performed as described previously (Bratt and Clavell, 1972). Briefly, the allantoic fluids of the rescued viruses were centrifuged with 500 × g for 20 min, and the supernatants were diluted to the same hemagglutination unit/ml (HAU/ml). Five hundred microliters of the diluted virus sample was mixed with 1 ml of 1% CRBC suspension and incubated on ice for 20 min. Then the CRBCs were centrifuged at 500 × *g* for 3 min, washed and resuspended with 0.5 ml PBS. After 1 h incubation at 37°C, tubes were centrifuged for 5 min at 200 × *g*. Subsequently, supernatant fluids were added to 96-well plates and the hemoglobin contents were measured at 549 nm with a Spectramax M5 ELISA reader (Molecular Devices). Positive and negative controls were 0.03 M NH_4_OH and PBS, respectively. The hemolytic values for each virus were expressed as percentages of the values for parental virus rSG10 and rLaSota at 2^8^ HAU/ml, which were considered to be 100%.

### Data Analysis

All data was analyzed using Prism 6.0 (GraphPad Software Inc., San Diego, CA, United States). Statistical differences among different groups were performed using the analysis of variance method followed by Tukey’s test. Statistical significance was set at ^∗^*p* < 0.05, ^∗∗^*p* < 0.01, and ^∗∗∗^*p* < 0.001.

## Results

### Recovery and Identification of Recombinant Chimeric Viruses

To investigate the impact of the different origins of HN proteins in NDV virulence, the complete HN ORF of strains BJ, LaSota, HB and Yucaipa was inserted into the rSG10 backbone in place of the corresponding rSG10 sequence. Likewise, the complete HN ORF of rLaSota was replaced with the corresponding ORF of strains SG10, BJ, HB and Yucaipa. The supernatants from transfected BSR T7/5 cells were passaged twice in embryonated SPF chicken eggs. The HA positive allantoic fluids were used for the isolation of viral RNA, followed by sequence analysis of an RT-PCR fragment to ensure the presence of the intended HN gene. Seven chimeric viruses were recovered, but the full-length cDNA encoding the Yucaipa HN protein in the rLaSota backbone did not yield a viable virus (**Figure 1A**). The rescued viruses were successively passaged 10 times via 9-day-old SPF eggs to further determine their stability. The sequencing results of the whole HN genes indicated that the seven rescued viruses were stable. Therefore, these seven chimeric viruses of the 10^th^ generations, together with their parental viruses (rSG10 and rLaSota), were used in subsequent experiments. The HN proteins in parental and chimeric viruses were analyzed by Western blotting using polyclonal anti-SG10 and anti-Yucaipa serum. This confirmed that the correct HN protein was present (**Figure 1A**).

### Growth of Chimeric Viruses in DF-1 Cells

The multicycle replication of chimeric and parental viruses was evaluated in DF1 cells (**Figure 1B**). The rSG10 constructs bearing the HN genes of strains BJ, LaSota, HB and Yucaipa showed decreased replication compared with parental rSG10. Differences in the growth kinetics of the viruses were observed after 24 hpi. The replication kinetics of rSG10-BJHN were slightly lower than rSG10, whereas rSG10-YuHN showed significantly decreased viral yields than the parental virus (*p* < 0.001 at 24 hpi; *p* < 0.01 at 36–72 hpi). rSG10-LaHN and rSG10-HBHN replicated to titers that were intermediate between the extremes of rSG10 and rSG10-YuHN.

When the growth of strains rLaSota-SGHN, rLaSota-BJHN, rLaSota-HBHN was compared with that of the parental rLaSota, rLaSota-SGHN and rLaSota-BJHN showed an obvious increase at 24 and 36 hpi (*p* < 0.05). rLaSota and its chimeric viruses grew to similar titers after 32 hpi. These results show the relevance of HN to virus growth *in vitro*, indicating that the HN protein could affect the growth of NDV, and the origin of the HN protein has a discernible impact.

### Virulence Assessment of Chimeric Viruses

Virulence of the seven chimeric viruses along with their respective parental viruses was evaluated by the MDT, the ICPI and the IVPI tests. The MDT values of rSG10 and rLaSota were 42 h and >90 h, and these viruses had ICPI scores of 1.93 and 0.00, and IVPI values of 2.79 and 0.00, respectively (**Table 1**). The chimeric viruses on the rSG10 background resulted in decreased virulence compared with parental rSG10. The MDT/ICPI/IVPI values were as follows: rSG10-BJHN, 50.4h/1.86/2.67; rSG10-LaHN, 57.6h/1.76/2.38; rSG10-HBHN, 55.2h/1.81/2.64; rSG10-YuHN, 72h/1.58/1.91, respectively. These results show that transfer of the Yucaipa HN protein into rSG10 had the greatest effect on decreasing virulence, altering the viral pathotype from velogenic to borderline mesogenic.

**Table 1:** Pathogenicity of the parental and recombinant viruses.

Virus	MDT (h)^a^	ICPI score^b^	IVPI score^c^
rSG10	42.0	1.93	2.79
rSG10-BJHN	50.4	1.86	2.67
rSG10-LaHN	57.6	1.76	2.38
rSG10-HBHN	55.2	1.81	2.64
rSG10-YuHN	72.0	1.58	1.91
rLaSota	>90	0.00	0.00
rLaSota-SGHN	>90	0.63	0.58
rLaSota-BJHN	>90	0.51	0.41
rLaSota-HBHN	>90	0.13	0.20


*^a^The minimum lethal dose of virus required to kill all the embryonated chicken eggs inoculated with virus by the allantoic route. Mean death time (MDT) pathotype definition: virulent strains, <60 h; moderately virulent strains, 60–90 h; avirulent strains, >90 h. ^b^Determined by inoculating groups of 10 one-day-old SPF chicks with fresh infective allantoic fluid via the intracerebral route. Intracerebral pathogenicity index (ICPI) definition: virulent strains, 1.5–2.0; moderately virulent strains, 0.7–1.5; avirulent strains, 0.0–0.7. ^c^Determined by inoculating groups of 10 six-week-old SPF chickens with fresh infective allantoic fluid via the intravenous route. Intravenous pathogenicity index (IVPI) definition: velogenic strains yield values greater than 2.0, while avirulent strains approach 0.0.*

Chimeric viruses on the rLaSota background showed increased virulence compared with parental rLaSota. The MDT/ICPI/IVPI values were as follows: rLaSota-SGHN, >90 h/0.63/0.58; rLaSota-BJHN, >90 h/0.51/0.41; rLaSota-HBHN, >90 h/0.13/0.20, respectively (**Table 1**). These findings show that transfer of the SG10 HN protein into rLaSota had the greatest effect on increasing virulence, resulting in a change in viral pathotype from lentogenic to borderline mesogenic. Thus, in both series, the virulence was affected by transfer of the HN gene.

### Replication and Pathogenicity of Chimeric Viruses in 3-week-old SPF Chickens

**FIGURE 2:**
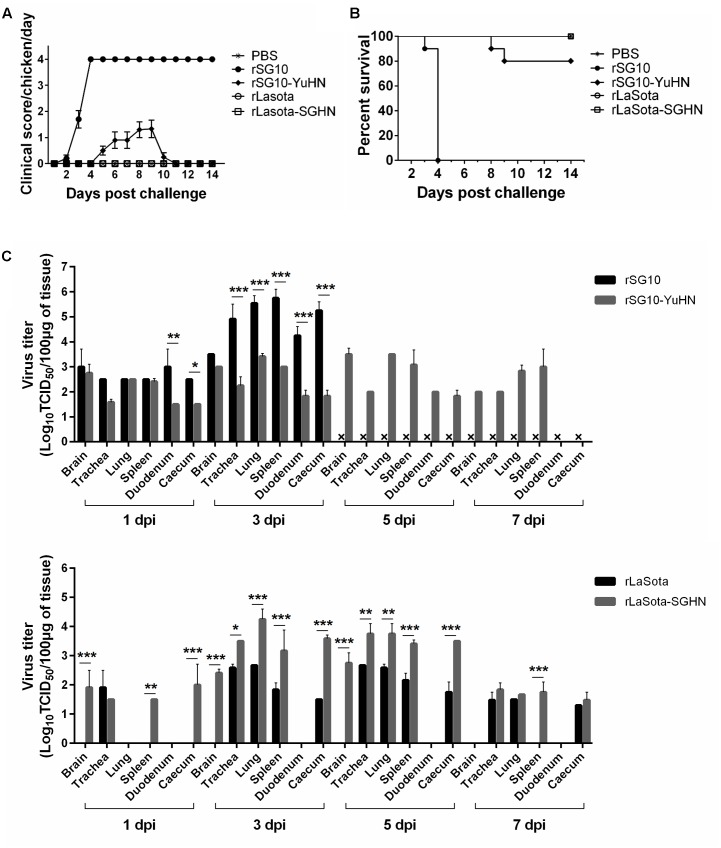
Clinical scores, survival rate, and replication of parental and chimeric viruses in 3-week-old chickens. Groups of 20 (10 for sampling and 10 for clinical observation) 3-week-old SPF chickens were inoculated with 10^5^ EID_50_ of rSG10, rSG10-YuHN, rLaSota, and rLaSota-SGHN through the natural route of infection. **(A)** Clinical signs in infected chickens were scored daily based on 10 birds per group (0, healthy; 1, sick; 2, wing drop/paralysis/torticollis/incoordination; 3, prostration; 4, dead). The daily mean scores for per group are shown. **(B)** Survival of 3-week-old SPF chickens inoculated with the parental and chimeric viruses based on 10 birds per group. **(C)** Replication of parental and chimeric viruses in 3-week-old chickens. The inoculated birds were sacrificed at 1, 3, 5, 7 dpi, the indicated tissues collected and virus titers determined by indirect immunofluorescence in DF-1 cells. The symbol “ × ” indicates no chickens survived for detection of virus. Asterisks indicate the significance of the difference between the chimeric and parental viral titers. ^∗^*p* < 0.05, significant; ^∗∗^*p* < 0.01, very significant; ^∗∗∗^*p* < 0.001, extremely significant.

To further evaluate the pathogenicity of two chimeric viruses (rSG10-YuHN and rLaSota-SGHN) and their parental viruses (rSG10 and rLaSota), 3-week-old chickens were inoculated with 10^5^ EID_50_ of virus per bird via the oculonasal route. All chickens infected with the parental rSG10 virus showed clinical signs at 3 dpi and 100% mortality at 4 dpi. At 5 dpi, clinical signs were observed in chickens infected with rSG10-YuHN. One chicken was found dead in the rSG10-YuHN group at both 8 and 9 dpi. All chickens infected with rSG10-YuHN began to recover at 10 dpi, and the clinical score was much lower than with rSG10. No obvious clinical signs were observed in any birds inoculated with rLaSota and rLaSota-SGHN (**Figures 2A,B**).

At 1, 3, 5, and 7 dpi, two chickens from each group were sacrificed and tissue samples were collected for virus titration (**Figure 2C**). For rSG10 and rSG10-YuHN, all sampled tissues in the rSG10-infected chickens showed high viral titers at 3 dpi. However, the virus titers in trachea, lung, spleen, duodenum and caecum were significantly lower in rSG10-YuHN-infected chickens than in chickens inoculated with rSG10 (*p* < 0.001), indicating a decreased replication ability of rSG10-YuHN compared with the parental virus. For rLaSota and rLaSota-SGHN, rLaSota could replicate in trachea and occasionally in lung, spleen and caecum, while rLaSota-SGHN showed an increased replication ability in a wide range of organs. rLaSota-SGHN replicated to moderate or high titers in trachea, lung, spleen and caecum, and showed significant differences (*p* < 0.001 or *p* < 0.01 for individual organs) compared with rLaSota.

**FIGURE 3:**
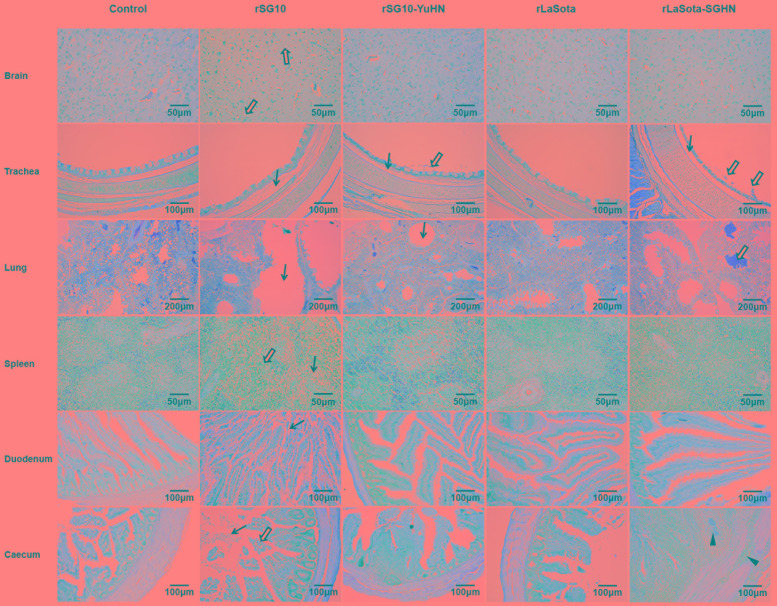
Tissue histopathology of inoculated 3-week-old chickens. Chickens were infected oculonasally with chimeric virus rSG10-YuHN, chimeric virus rLaSota-SGHN or parental viruses (rSG10 and rLaSota). Birds were sacrificed at 3 dpi and tissues were fixed with formalin, sectioned and stained with hematoxylin and eosin. The brain showed loose tissue and vacuolation (empty arrows). The trachea had interstitial broadening, edema (black arrows) and slight exfoliation of tracheal mucosa epithelium (empty arrows). The lung had bronchiectasis, emphysema (black arrows) and hemorrhage (empty arrows). The spleen had lymphocyte necrosis (black arrows), a large number of infiltrating inflammatory cells and multifocal confluent coagulative necrosis (empty arrows). The duodenum had mucosal epithelium exfoliation (black arrows). The caecum had mucosal epithelium exfoliation (black arrows), increased goblet cells in the mucosal epithelium (empty arrow) and inflammatory cell infiltration of the mucosa lamina propria and muscularis mucosa (black triangles).

Two chickens from each group were sacrificed at 3 dpi for histopathological analysis (**Figure 3**). The rSG10 caused the moderate to severe histological changes in the sampled tissues: loose brain tissue with vacuolation (empty arrows); interstitial broadening, edema (black arrow) in the trachea; bronchiectasis and emphysema (black arrow) in the lung; excessive inflammatory cell infiltration, multifocal confluent coagulative necrosis (empty arrow) and lymphocyte necrosis (black arrow) in the spleen; mucosal epithelium exfoliation (black arrow) in the duodenum; mucosal epithelium exfoliation (black arrow), increased goblet cells in the mucosal epithelium (empty arrow) of the caecum. However, the chimera rSG10-YuHN exhibited decreased virulence with mild lesions in the trachea and lung: interstitial broadening, edema (black arrow) and slight exfoliation of the tracheal mucosa epithelium (empty arrow), bronchiectasis and emphysema (black arrow) in the lung, but no obvious changes in the brain, spleen, duodenum or caecum compared with the parental rSG10 virus.

For the chimera rLaSota-SGHN and its parental virus rLaSota, no obvious histological changes were found in sampled tissues of the group inoculated with rLaSota. On the contrary, rLaSota-SGHN caused increased virulence with mild or moderate histological changes in trachea, lung and caecum: interstitial broadening, edema (black arrow) and slight exfoliation of the tracheal mucosa epithelium (empty arrows), hemorrhage (empty arrow) in the lung, and inflammatory cell infiltration to the mucosa lamina propria and muscularis mucosa (black triangles) in the caecum (**Figure 3**). These results demonstrate that the HN protein affects replication and pathogenicity of NDV in chickens, and the extent of the impact is related to the origin of the HN protein.

### HAd and NA Activities of Chimeric Viruses

**FIGURE 4:**
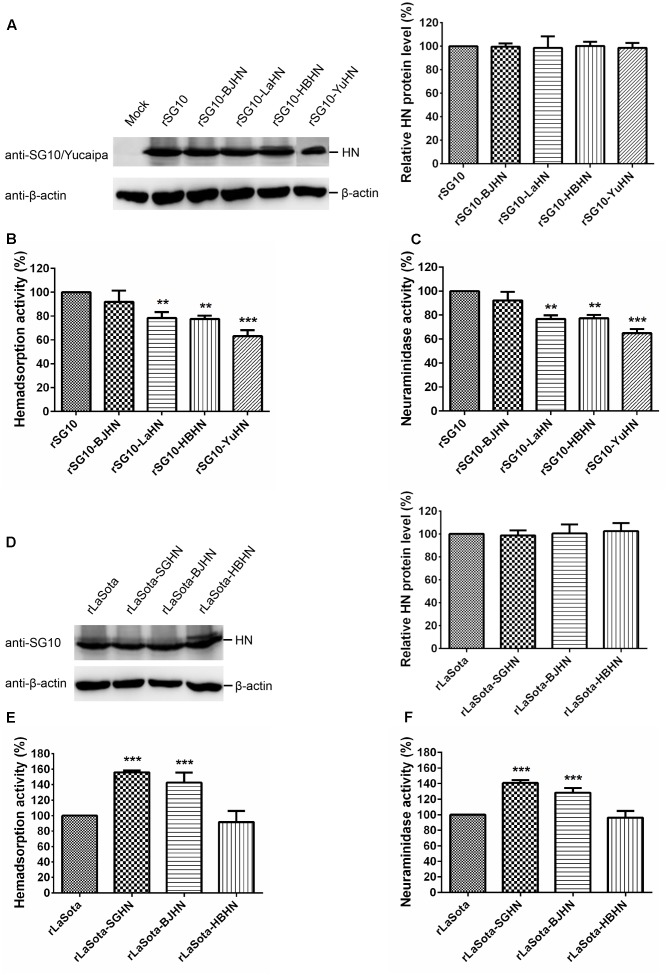
HAd and NA activities of parental and chimeric viruses. **(A,D)** The expression amount of each HN protein was determined by Western blotting using anti-SG10 or anti-Yucaipa serum. HAd **(B,E)** and NA activities **(C,F)** were examined in Vero cells infected with virus at an MOI of 0.1. HN protein expression level, HAd and NA activities of these viruses are expressed as percentages of the values for parental viruses (rSG10 and rLaSota), which were set at 100%. Each bar represents the mean and standard deviation of three independent experiments. Asterisks indicate the significance of the differences between the biological activity of a chimeric virus and that of the parental virus. P values were calculated with Tukey’s test (95% confidence levels). ^∗∗^*p* < 0.01, very significant; ^∗∗∗^*p* < 0.001, extremely significant.

To determine whether the different origins of HN proteins have a differing influence on the biological activities of NDV in cultured cells, the mutant and parental viruses were determined for their HAd and NA activities (**Figure 4**). The HN protein expression of recombinant viruses was measured in Vero cells, and indicated that HN expression in the chimeric viruses was similar to that of the respective parental HN (**Figures 4A,D**). The biological activities of each chimeric virus were analyzed and calculated as a percentage of that of the respective parental virus (rSG10 and rLaSota), whose biological activities were deemed to be 100%. **Figures 4B,C** show that HAd and NA activities of rSG10-BJHN were slightly lower than the parental virus rSG10, whereas significant reductions were observed in both HAd and NA activities for chimeras rSG10-LaHN (78 and 76%, respectively; *p* < 0.01), rSG10-HBHN (77 and 77%, respectively; *p* < 0.01), and rSG10-YuHN (63 and 65%, respectively; *p* < 0.001). For the chimeric viruses with the rLaSota backbone, rLaSota-SGHN and rLaSota-BJHN showed a 56 and 42% increase, respectively, in HAd activity, and 40 and 28% increase, respectively, in NA activity over those of the rLaSota parent (*p* < 0.001). HAd and NA activities of rLaSota-HBHN were similar to that of the parent virus (**Figures 4E,F**). These results reiterate the importance of the HN protein in the attachment and NA functions of NDV in the context of viral infection. Furthermore, these data demonstrate that the different origins of HN proteins influence the HAd and NA activities of NDV to varying degrees.

### Fusion Indices and Hemolytic Activities of the Recombinant Viruses

**FIGURE 5:**
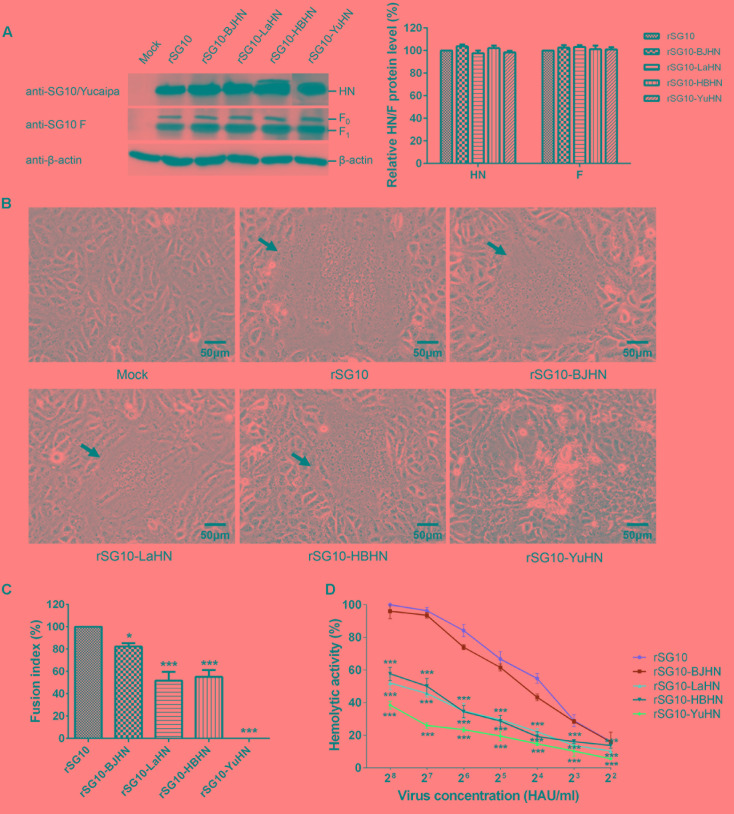
Syncytium formation and hemolytic activities of rSG10 and its chimeric viruses. **(A)** The levels of each HN and F (F_0_ and F_1_) proteins were quantitated by Western blotting using sera specific to the individual SG10, Yucaipa or anti-SG10 F protein rabbit polyclonal antiserum, as indicated. The expression of HN and F proteins are expressed as percentages of the values for rSG10. **(B)** Syncytium formation was induced by viral infection of Vero cells. Black arrows indicate syncytia. Bar indicates 50 μm. **(C)** The fusion index values of chimeric viruses were calculated as the ratio of the total number of nuclei to the number of cells in which the nuclei were observed and expressed relative to the value for rSG10 (100%). **(D)** The hemolytic activities for chimeric viruses were expressed as percentages of the value for rSG10 at 2^8^ HAU/ml, which was set at 100%. Each bar represents the mean and standard deviation of three independent experiments. Asterisks indicate statistically significant differences. *P*-values were calculated with Tukey’s test (95% confidence levels). ^∗^*p* < 0.05, significant; ^∗∗∗^*p* < 0.001, extremely significant.

**FIGURE 6:**
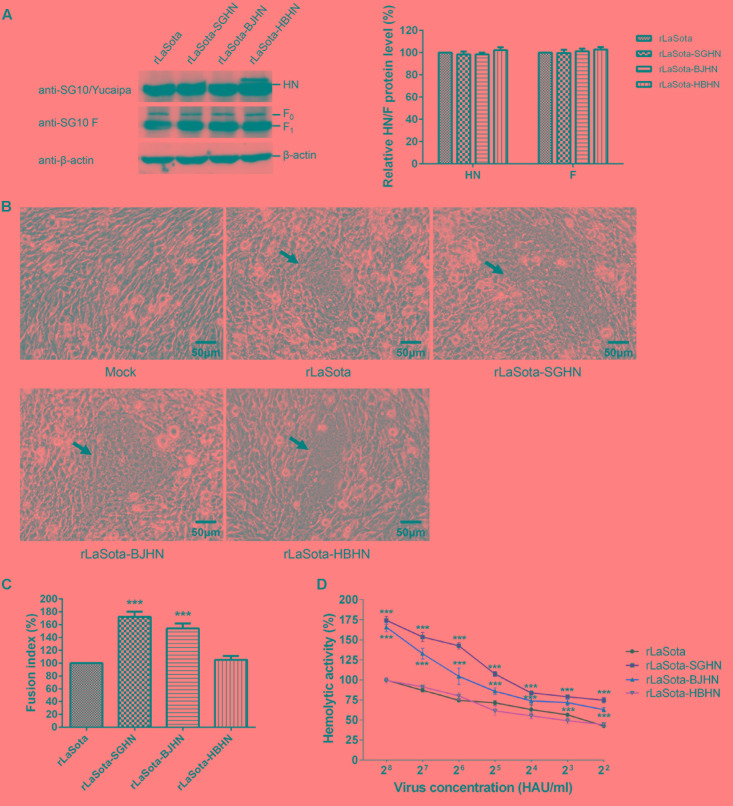
Syncytium formation and hemolytic activities of rLaSota and its chimeric viruses. **(A)** The levels of each HN and F (F_0_ and F_1_) proteins were quantitated by Western blotting using sera specific to the individual SG10, Yucaipa and anti-SG10 F protein rabbit polyclonal antiserum, as indicated. The expression of HN and F proteins are expressed as percentages of the values for rLaSota. **(B)** Syncytium formation was induced by viral infection of DF-1 cells with 5 μg/ml TPCK-treated trypsin. Black arrows indicate syncytia. Bar indicates 50 μm. **(C)** The fusion index values of chimeric viruses were calculated as the ratio of the total number of nuclei to the number of cells in which the nuclei were observed and expressed relative to the value for rLaSota (100%). **(D)** The hemolytic activities for chimeric viruses were expressed as percentages of the value for rLaSota at 2^8^ HAU/ml, which was set at 100%. Each bar represents the mean and standard deviation of three independent experiments. Asterisks indicate statistically significant differences. *P*-values were calculated with Tukey’s test (95% confidence levels). ^∗∗∗^*p* < 0.001, extremely significant.

To further examine whether the substitution of the different HN proteins affected the fusogenic promotion activity of the virus, we measured fusion indices of rSG10 and its chimeric viruses in Vero cells, rLaSota and its chimeric viruses in DF-1 cells, and the hemolytic activities of all experimental viruses (**Figures 5**, **6**). Western blot analysis indicated that the HN and F (F_0_ and F_1_) expressions of the derivatives of rSG10 and rLaSota were similar to that of respective parental virus (**Figures 5A**, **6A**). The syncytia induced by rSG10-BJHN, rSG10-LaHN and rSG10-HBHN were markedly smaller than those induced by rSG10, while syncytium was not induced by rSG10-YuHN (**Figure 5B**). For the chimeric viruses with the rSG10 backbone, the fusion indices (relative to rSG10, as 100%) were as follows: rSG10-BJHN, 82%; rSG10-LaHN, 52%; rSG10-HBHN, 55%; rSG10-YuHN, 0% (**Figure 5C**). Conversely, for the chimeric viruses with the rLaSota backbone, the size of syncytia induced by rLaSota-SGHN and rLaSota-BJHN were increased significantly compared with those induced by parental rLaSota (**Figure 6B**). The fusion indices (relative to rLaSota, as 100%) were as follows: rLaSota-SGHN, 172%; rLaSota-BJHN, 154%; rLaSota-HBHN, 105% (**Figure 6C**). These results showed that the origin of the HN protein had differing effects on the fusogenic promotion activity of the virus.

Subsequently, the hemolytic activities of recombinant viruses were tested at virus concentrations ranging from 2^2^ to 2^8^ HAU/ml. The hemolytic values are expressed as percentages of the values for parental viruses rSG10 and rLaSota at 2^8^ HAU/ml, which were considered to be 100%. For rSG10 derivatives bearing the HN genes of strains BJ, LaSota, HB and Yucaipa, the hemolytic activity of rSG10-BJHN was slightly lower than parental virus rSG10, whereas rSG10-LaHN, rSG10-HBHN and rSG10-YuHN were significantly decreased compared with rSG10 (*p* < 0.001). Furthermore, rSG10-YuHN showed the greatest reduction in hemolytic activity of all the mutants (**Figure 5D**). As shown in **Figure 6D**, for rLaSota derivatives bearing the HN genes of strains SG10, BJ and HB, the hemolytic activity of rLaSota-HBHN was similar to that of rLaSota, but rLaSota-SGHN and rLaSota-BJHN were significantly increased compared with parental rLaSota (*p* < 0.001). These results indicate that the hemolytic activity of NDV is influenced by HN, and the origin of this protein can influence hemolysis and fusion of the virus.

### Biological Activities of HN Proteins of Different Origins at the Protein Level

**FIGURE 7:**
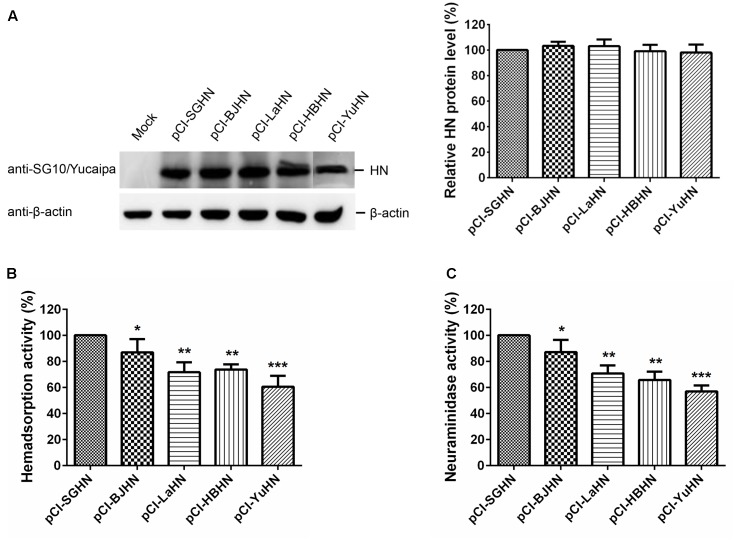
HAd and NA activities of the different origins of HN proteins at the protein level. **(A)** The relative expression of each HN protein was examined by Western blot analysis at 24 h post-transfection of Vero cells by using anti-SG10 or anti-Yucaipa serum. HAd **(B)** and NA activities **(C)** were measured in Vero cells transfected with the 0.5 μg each of pCI-HN plasmids. The HN protein level, HAd and NA activities of the HN proteins of different origins are expressed as percentages of the values for expressed SGHN protein, which was considered to be 100%. Each bar represents the mean and standard deviation of three independent experiments. Asterisks indicate statistically significant differences. ^∗^*p* < 0.05, significant; ^∗∗^*p* < 0.01, very significant; ^∗∗∗^*p* < 0.001, extremely significant.

Next, we investigated the HAd, NA and fusogenic promotion activities of the various HN proteins at the protein level, using HN expression plasmids (pCI-SGHN, pCI-BJHN, pCI-LaHN, pCI-HBHN and pCI-YuHN). The results were consistent with those of rSG10 derivatives bearing the HN genes of strains BJ, LaSota, HB and Yucaipa at the virus level; expression of the different HN proteins was similar in Vero cells (**Figure 7A**). In this case, BJHN, LaHN, HBHN and YuHN proteins showed an 87, 72, 74, and 61% decrease, respectively, in HAd activity and 87, 71, 66, 57% decrease, respectively, in NA activity compared with the SGHN protein (**Figures 7B,C**) (*p* < 0.05, *p* < 0.01, or *p* < 0.001, depending on HN origin). These results demonstrate that HAd and NA activities of HN proteins of different origins varied at the protein level, with SGHN showing the highest HAd and NA activities, while receptor-binding ability and NA activity of YuHN protein were lowest.

**FIGURE 8:**
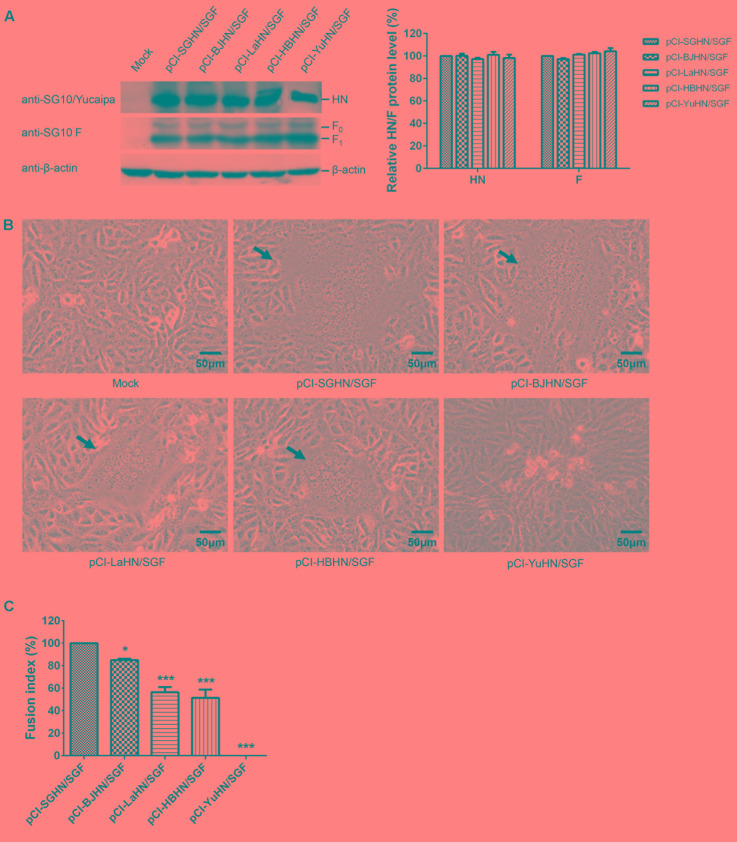
Syncytium formation in Vero cells co-transfected with expressed plasmids. **(A)** The relative expression of each HN or F (F_0_ and F_1_) protein was measured by Western blotting using sera specific to the individual SG10, Yucaipa and anti-SG10 F protein rabbit polyclonal antiserum. **(B)** Syncytium formation was induced by co-transfection with 1 μg each of pCI-HN and pCI-SGF. Black arrows indicate syncytia. Bar indicates 50 μm. **(C)** The fusion index values were calculated as the ratio of the total number of nuclei to the number of cells in which the nuclei were observed. All values are expressed relative to the value for pCI-SGHN/SGF (100%). Each bar represents the mean and standard deviation of three independent experiments. Asterisks indicate statistically significant differences. P values were calculated with Tukey’s test (95% confidence levels). ^∗^*p* < 0.05, significant; ^∗∗∗^*p* < 0.001, extremely significant.

**FIGURE 9:**
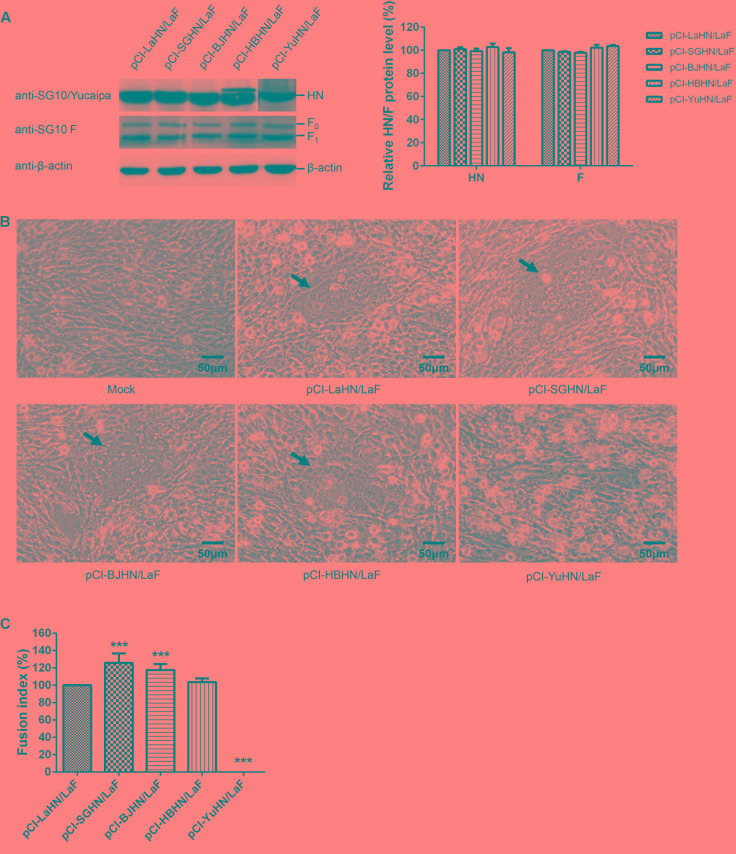
Syncytium formation in DF-1 cells co-transfected with expressed plasmids. **(A)** The amount of each HN or F (F_0_ and F_1_) protein expressed was examined by Western blot analysis using sera specific to the individual SG10, Yucaipa and anti-SG10 F protein rabbit polyclonal antiserum. **(B)** Syncytium formation was induced by co-transfection with 1 μg each of pCI-HN and pCI-LaF. Black arrows indicate syncytia. Bar indicates 50 μm. **(C)** The fusion index values were calculated as the ratio of the total number of nuclei to the number of cells in which the nuclei were observed. All values are expressed relative to the value for pCI-LaHN/LaF (100%). Each bar represents the mean and standard deviation of three independent experiments. Asterisks indicate statistically significant differences. *P*-values were calculated with Tukey’s test (95% confidence levels). ^∗∗∗^*p* < 0.001, extremely significant.

Furthermore, the fusogenic promotion activities at the protein level of the different HN proteins were evaluated in Vero (**Figure 8**) and DF-1 cells (**Figure 9**) using HN protein expression plasmids (pCI-SGHN, pCI-BJHN, pCI-LaHN, pCI-HBHN, and pCI-YuHN) and F protein expression plasmids (pCI-SGF and pCI-LaF). The result had the same trend as at the viral level. The HN proteins of different origins showed significantly decreased fusion indices compared with co-transfection of pCI-SGHN and pCI-SGF under similar HN and F expression. The order of reduction (relative to SGHN/SGF, 100%) was BJHN/SGF (85%; *p* < 0.05), LaHN/SGF (56%; *p* < 0.001), HBHN/SGF (51%; *p* < 0.001) and YuHN/SGF (0%; *p* < 0.001) (**Figure 8C**). As for the co-expression of the different HN proteins and LaSota F protein (LaF) in DF-1 cells, syncytium formation of SGHN/LaF and BJHN/LaF showed a 26 and 17% increase, respectively, over LaHN/LaF (*p* < 0.001). The fusion index of HBHN/LaF was roughly identical to the LaHN/LaF value. Syncytia were not induced by co-expression of YuHN and LaF (**Figures 9B,C**). These results indicate that fusogenic promotion activities of the different HN proteins are different at the protein level, as SGHN protein showed the highest fusogenic promotion ability, while YuHN protein was unable to induce syncytium formation.

## Discussion

The virulence of different NDVs shows great variation within the single serotype and the molecular basis for this is not fully understood. Previous studies have shown that the amino acid sequence at the F protein cleavage site is a major determinant, but multiple proteins are associated with NDV virulence (Nagai et al., 1976; Toyoda et al., 1987; Glickman et al., 1988; Peeters et al., 1999; Huang et al., 2003; Panda et al., 2004; De Leeuw et al., 2005; Rout and Samal, 2008; Dortmans et al., 2010; Paldurai et al., 2014; Yu et al., 2017). In this study, exchange of the HN gene from a velogenic or lentogenic NDV backbone strain and other strains with different virulence was performed to reveal the exact role of the HN protein in NDV virulence. This is the first comprehensive and systematic study of the HN protein and NDV virulence.

The HN protein of NDV plays multiple roles during host viral infection, including receptor binding, neuraminidase and fusion promotion activities (Crennell et al., 2000; Morrison, 2001; Melanson and Iorio, 2004). Some studies have indicated that the HN protein is also an important determinant of NDV virulence, but great variation exists between the results of different studies (Huang et al., 2004; De Leeuw et al., 2005; Kim et al., 2011; Adu-Gyamfi et al., 2016). Differences in these studies may be associated with the different NDV strains involved. Kim et al. (2011) investigated the role of the F and HN proteins by designing chimeric viruses in which the F and HN or their ectodomains were exchanged individually or together between the moderately virulent NDV strain Beaudette C and the avirulent APMV-2 strain Yucaipa. They recovered only three viruses: the recombinant APMV-2 strain Yucaipa (rAPMV-2) which contained the NDV F protein, rAPMV-2 containing both the F and HN ectodomains of NDV, and rNDV which contained both the F and HN ectodomains of APMV-2. In this study, we successfully recovered the recombinant NDV strain rSG10, containing the APMV-2 Yucaipa HN glycoprotein in place of its own. The construct could be stably passaged despite a low amino acid sequence homology of 33.4% between NDV SG10 and APMV-2 Yucaipa, indicating that HN proteins have a level of compatibility among different APMVs. However, viable virus could not be recovered from the chimeric NDV LaSota cDNA that contained the APMV-2 Yucaipa HN gene after many attempts. The virulent strains with stronger infectivity tended to be favorable for viral rescue and this may explain the failure to recover avirulent chimeras.

Although disparities in tissue tropism and *in vivo* pathogenicity can be observed in some viruses, it is quite conceivable that viral tissue tropism is an important factor determining the virulence and broad tropism results in more severe pathogenicity or higher virulence for many viruses (Haller et al., 1995; Dortmans et al., 2011; Meliopoulos et al., 2014; Chhabra et al., 2016). For NDV, the HN protein was considered to play a major role in the tissue tropism (Huang et al., 2004). We systematically determined the tissue tropism of NDV by exchanging HN proteins between a velogenic (rSG10) or lentogenic (rLaSota) NDV strain and several other strains of different virulence. Our results showed that the viral tissue tropism was dependent on the origin of HN protein. The HN protein derived from the lentogenic virus within a backbone of a virulent strain exhibited a reduced tissue tropism and vice versa. These findings are in line with a previous study (Huang et al., 2004) and were slightly different from the results of several researchers (Oldoni et al., 2005; Wakamatsu et al., 2006). We think it is more reasonable that the HN protein derived from a virulent virus could increase and conversely that derived from a lentogenic virus can decrease the tissue tropism of the viruses. Generally, virulent strains are more infectious and can evidently enhance the biological activity of the chimeric virus (Dortmans et al., 2010; Paldurai et al., 2014; Jin et al., 2016).

The HN protein of NDV plays an important role in viral infection because it possesses the receptor recognition activity, while viral infection is initiated by attachment of the virion to the sialic acid-containing receptors on the surface of the target cell. HN protein can also promote fusion activity via its interaction with the F protein and act as an NA by removing sialic acid from progeny virus particles to prevent viral self-aggregation, aiding virus infection (Mirza et al., 1994; Huang et al., 2004; Melanson and Iorio, 2004; Heiden et al., 2014; Liu B. et al., 2015; Jin et al., 2016). Our results showed that HN proteins of the five selected NDV strains in this study had markedly different HAd, NA and fusogenic promotion activities at the protein level. Accordingly, the chimeric viruses also exhibited different biological activities, especially in receptor recognition and NA activities, which may be the major reason for the difference in viral replication among different chimeric viruses *in vitro* and *in vivo*. As we know, there is a positive correlation between viral pathogenicity and its replication efficiency (Bankamp et al., 2002; Dortmans et al., 2011; Jin et al., 2016). We think that the differing biological activity of HN proteins results in demonstration of different pathogenicities of the chimeric viruses in chickens. Furthermore, the HAd, NA and fusogenic promotion activities of HN proteins also influence the replication ability of NDV and thus the virulence.

Membrane fusion is necessary for NDV entry into host cells. The F protein is directly involved in membrane fusion and is a major determinant of NDV virulence, whereas the HN protein possesses fusion promotion activity by its interaction with the F protein and is therefore another determinant of viral virulence (Kim et al., 2011; Jin et al., 2016). Some recently published data showed that the stalk domains of the HN protein could promote the fusion event mediated by the F protein (Bose et al., 2012, 2014; Sun et al., 2015). The HN stalk domains included two relatively conserved heptad repeats (HR), with HR1 comprising residues 79–88 and HR2 comprising residues 96–110. The HR1 and HR2 regions are separated by an intervening region (IR) from residues 89–95 (Melanson and Iorio, 2004; Iorio et al., 2009; Yuan et al., 2015). Liu B. et al. (2015) found that A89Q and L94A mutations in the IR region weakened the fusion promotion activity of the HN protein. In this study, the rSG10 derivative rSG10-YuHN bearing the HN protein gene of rAPMV-2 Yucaipa exhibited significantly decreased fusogenic promotion activity (*p* < 0.001). Vero cells could not form syncytium after infection with rSG10-YuHN. Sequence analysis of the HN protein revealed that there was low amino acid sequence identity (33.4%) between the APMV-2 Yucaipa and NDV SG10 strains, the residues at position 89 and 94 of IR region were also different between Yucaipa and other four viruses, which could be a possible reason for the decline of the fusion activity and virulence of the recombinant virus rSG10-YuHN. It is also reported that the conformation of the HN stalk domain in other paramyxoviruses could influence the interaction of HN protein with F protein in the cell-cell fusion (Tsurudome et al., 2015). Whether this notion is applicable to NDV HN proteins needs to be further investigated.

## Conclusion

Here we have comprehensively and systematically studied the correlation between the HN protein and NDV virulence. Using HN exchanges between a velogenic or lentogenic NDV strain and other strains of different virulence, we have demonstrated that alteration of the balance between receptor recognition, NA and fusion promotion activities causes changes in tissue tropism, replication and pathogenicity of the virus, which was closely related to the origin of the HN protein. Our study further highlights the importance of the HN glycoprotein in modulating the virulence of NDV. Further studies should concentrate on how to regulate the balance of the biological activities of the HN protein, which may provide a more complete understanding of the virulence of NDV and a more effective preventive approach for the disease.

## Author Contributions

Conceived and designed the experiments: J-hJ, J-lC, Z-rH, G-zZ. Performed the experiments: J-hJ, J-lC, Z-rH, Y-cR, X-hY, YS, H-mY, Y-lY, and TL. Analyzed the data: J-hJ, J-lC, Z-rH, X-hY, and G-zZ. Contributed reagents/materials/ analysis tools: G-zZ. Wrote the paper: J-hJ, J-lC, Z-rH, and G-zZ.

## Conflict of Interest Statement

The authors declare that the research was conducted in the absence of any commercial or financial relationships that could be construed as a potential conflict of interest.
